# Willingness to Consult a Veterinarian on Physician’s Advice for Zoonotic Diseases: A Formal Role for Veterinarians in Medicine?

**DOI:** 10.1371/journal.pone.0131406

**Published:** 2015-08-03

**Authors:** Rick Speare, Diana Mendez, Jenni Judd, Simon Reid, Saul Tzipori, Peter D Massey

**Affiliations:** 1 Tropical Health Solutions, Townsville, Queensland, Australia; 2 College of Public Health, Medical and Veterinary Sciences, James Cook University, Townsville, Queensland, Australia; 3 Division of Tropical Health and Medicine, James Cook University, Townsville, Australia; 4 School of Population Health, University of Queensland, Brisbane, Australia; 5 Cummings School of Veterinary Medicine, Tufts University, Boston, United States of America; 6 Hunter New England Population Health, Tamworth, Australia; Kent State University, UNITED STATES

## Abstract

Physicians appear to find zoonotic diseases a challenge and consider that this topic belongs more to the veterinary profession. However, veterinarians have no formal role in clinical medicine. Data were collected as part of the Queensland Social Survey 2014 to determine the willingness of the public, if diagnosed with a zoonotic disease, to consult a veterinarian on the advice of a physician. Self-reported willingness to consult with a veterinarian at the respondent’s own expense was 79.8% (95% CI: 81.96%-77.46%) (976/1223). If the cost was funded by Medicare, the Australian public health insurance scheme, 90.7% (95% CI: 92.18%-88.92%) (1109/1223) would be willing to consult a veterinarian. Therefore, a large majority of Australian residents would be willing to consult with a veterinarian on the advice of their physician if they had a zoonotic disease. Does this indicate a possible new role for veterinarians under Clinical One Health?

## Introduction

Zoonotic diseases, those infections acquired by humans from vertebrate animals, are an important component of the burden of communicable diseases [1,2]. In addition humanoses (previously called anthroponoses), infections acquired by animals from humans, are also being recognized more frequently; Influenza A H1N1 2009 illustrated this with humans infecting swine and poultry [3,4]. Once the spillover has occurred in either direction and, if the pathogen has become established in the new host species, spillback to the original host may be the next development. For example, methicillin resistant *Staphylococcus aureus*, which was exclusively a human pathogen, is now established in many domesticated animal species and is spilling back to infect humans [5,6]. Similarly, vaccinia virus in Brazil crossed from humans to cattle, became endemic and is now spilling back to cause disease in people [7]. The management of such diseases may be more effective using a One Health multiple-species approach.

The One Health approach aims to improve efficiency by having human, animal and environmental health disciplines working together in a transdisciplinary model and is endorsed by the World Health Organization, the World Organisation for Animal Health, Food and Agricultural Organization, and multiple bodies at international and national levels [8].

At the policy level One Health seems to be making headway although ecosystem health is rarely included [8]. Putting the One Health approach into practice has been a challenge, particularly at the clinical level [9,10]. At the coal face professions still remain relatively isolated by institutional and disciplinary barriers, resulting in professional silos [11–14]. Proponents of One Health appear to ignore potential efficiencies gained by veterinarians and physicians collaborating on improving clinical practice [10].

Although physicians diagnose and treat zoonotic diseases, they appear to find them challenging. Surveys indicate that physicians feel that veterinarians are the profession with much better knowledge and understanding of zoonoses [11,13]. This is likely to be more about risk mitigation strategies and ongoing risk of exposure than therapy. Physicians have suggested that veterinarians should join them in managing patients with zoonoses [11]. However, veterinarians have no formal role in the human health care system. This probably explains why the communication between the two professions about zoonoses is so limited. One survey found that just over half the veterinarians surveyed had never spoken to a physician about a zoonosis [15]. The outcome is that the profession thought to be the most expert in zoonoses is not involved in the management of individual patients [11,13]. If physicians need expert assistance to manage a patient with a zoonotic disease, they typically refer them to an infectious disease physician although there is no guarantee that this specialist is fully trained in the epidemiology, prevention and other aspects of zoonoses [11].

Individual physicians may have personal connections with veterinarians and use these to exchange information. However, physicians are not permitted to reveal identified clinical information to veterinarians under privacy regulations, which limits information exchange to anonymous generalities. Patients may also have specific questions about their disease for which they are unable to get accurate tailored advice from a physician or other health professionals.

Veterinarians may have a role in providing more accurate and comprehensive information on the epidemiology of the zoonotic disease a patient has; e.g., highlighting the importance of differentiating between animal, human and environmental sources of dermatophytoses in a patient that blames their pet kitten [16]. In addition veterinarians could assist in evaluating situation specific risk factors; e.g., advising a pig hunter with brucellosis due to *Brucella suis* how to decrease risks of reinfection and of acquiring other zoonoses such as Q fever and leptospirosis and also about possible transmission from his hunting dogs [17–20]. Veterinarians could also advise on risk-reduction strategies for the patient and their close contacts; e.g., salmonellas acquired from sheep farming in New Zealand and spread to family members on work clothes [21]. Advice could include strategies the patient can adopt by managing the disease in the source animals, particularly where balancing costs and benefits are important in decisions; e.g., helping a dairy farmer with leptospirosis to decide on whether to vaccinate her herd against leptospirosis to protect herself and other workers [22]. Veterinarians could also have a role in advising patients who may be at increased risk of zoonoses. This could include immunodeficient and immunosuppressed patients and pregnant women; e.g., explaining the importance of flea control in the pet cat to reduce the risk of bacillary angiomatosis due to *Bartonella henselae* in an AIDS patient [23]. Veterinarians could give advice that reduced risk at source as well along the chain of transmission [13]. Veterinarians and physicians could work together to identify whether sources of recurrent infections in a patient are animal or human; e.g., recurrent skin infections due to *Staphylococcus aureus* in several members of a family with pets and contact with livestock [24]. A formal role may also enable physicians themselves to obtain advice from the veterinarian about their clinical management of the case or to involve them in contributing to diagnostic decisions in cases that appear to be zoonotic but a diagnosis has not been confirmed. This interaction would arguably build capacity to deal with zoonoses in both professions.

Veterinarians could also play a role outside zoonotic diseases in advising patients that have suffered physical trauma from animals, particularly dog and cat bites and large livestock [25,26]. Pets are often a component in domestic violence cases and physicians may find veterinary involvement helpful [27–29].

Australia is similar to most developed nations in having well-functioning public and private health care systems. Universal health insurance is provided by Medicare, a system supported by the federal government [30]. Veterinarians on the other hand almost wholly work in the private sector, either for individually owned private practices or for veterinary companies [31]. In 2006 10% of Australian veterinarians were employed by government, mainly in policy and administrative roles [31].

Medicare provides support to all sectors of the health system, paying physicians either directly or indirectly on a fee for service basis. Under Medicare, physicians can also refer patients for a range of proscribed health services delivered by allied health professionals [30]. These services are limited to specific conditions and patients and clearly described in regulations.

One (of many) essential steps in involving veterinarians in the health care pathway is to gauge whether the public would see this as a valuable addition or not. Hence, the aim of this study was to explore the willingness of members of the Australian adult population to consult with a veterinarian if their primary physician recommended it to improve the management of their zoonotic disease. A secondary aim was to see if this willingness was sensitive to price.

## Methods

Data for this study were collected as part of the Queensland Social Survey (QSS) 2014, Australia, which is an annual state-wide survey conducted by the Population Research Laboratory in Central Queensland University’s (CQUni) Institute for Health and Social Science Research. Through a cost-sharing arrangement, QSS enables researchers and policy-makers to incorporate questions into the survey. Queensland is the second largest Australian state by land area, and the third most populous state. The QSS uses a computer-assisted telephone interviewing (CATI) system and trained interviewers to randomly sample households across Queensland, including metropolitan Brisbane (South East Queensland) and the rest of the state (Other Queensland). To permit the analysis of each area as a separate entity required a minimum sample size of 400 for each sub-region. To ensure a representative sample of the population in the QSS, households are randomly pre-determined to provide a male or female respondent; if a person of that sex was not available then the household was not included in the survey. The QSS 2014 consisted of a standardized introduction, specific questions incorporated by researchers and the University, and 37 demographic questions. The questions were pilot tested by trained interviewers in 68 randomly-selected households, with modifications to the questions guided by both responses from the pilot study subjects and feedback from the interviewers. Final interviewing was conducted between 29 July 2014 and 31 August 2014, between the hours of 10:30 am to 2:30 pm and 4:30 pm to 8:30 pm on weekdays, and between the hours of 12:00 pm and 4:00 pm on weekends. Two questions related to respondents’ anticipated compliance with a physician’s advice to consult with a veterinarian if they had the diagnosis of a disease acquired from an animal (zoonosis). The first question was: If your doctor diagnosed you with an infectious disease that had been acquired from an animal would you be willing to have an additional consultation with a veterinarian at your own expense to learn more about the disease if your doctor recommended it? For those who did not respond “yes”, a second question was asked: Would you be willing if the veterinarian consultation fee was funded under Medicare? Responses to the first question were recorded as “No, I would not consider doing this”; “No, I might consider it but wouldn't be willing to do it”; “Yes, I would be willing to do this”; “Don’t know” or “Unsure”. Responses to the second question were recorded as “Yes” or “No”.

Responses for age were categorized into the following categories: 18–24, 25–34, 35–44, 45–54, 55–64, 65–74, 75–84, 85 and above; and education levels were collapsed into six categories: primary, secondary, technical, tertiary or higher, None, and “don’t know”. Data was analyzed using SPSS (Statistical Package for Social Sciences, Version 22.0 Armonk, NY: IBM Corp.). Fisher’s exact test was used to compare participants who “would be willing to have an additional consultation with a veterinarian at their own expense” and those who “would be willing to do so if the consultation fee was funded under Medicare” with those who “would not be willing to either of the above or were unsure” by gender, age at last birthday, Australian citizenship status, level of education, work pattern in week prior, work status (full-time, part-time, causal), industry of work, employment status, gross income /week, combined household gross income/week, and type of area lived in (city, town, rural area, as per categories provided by QSS). When exact statistical results were not available directly through SPSS, a Monte Carlo simulation in SPSS was used to approximate exact results for Fisher’s test. Bivariate statistical analyses were performed with an alpha level of 0.05.

### Ethics Statement

This study was approved by the Human Ethics Review Panel at CQUni (H13/06-120). Verbal consent was provided by participants prior to commencement of the phone interview since written consent is impractical in a CATI survey. Consent was recorded electronically on a database. This procedure was approved by the CQUni Human Ethics Review Panel.

## Results

The QSS 2014 contacted or attempted to contact 3,438 households; 1,886 subjects declined participation, 165 households could not be contacted, and 153 were otherwise ineligible. Thus, the final sample for the QSS 2014 included 1,223 complete surveys (11 surveys only partially completed were not included); 814 from South East Queensland and 409 from Other Queensland, achieving the sample size for both regions. The sample was equally divided between males and females (50%). Younger people (aged 18–34 years) were under-represented in the sample and older people (aged > 55 years) were over-represented in the sample (Table 1). Index of dissimilarity was 28.1% [32,33].

**Table 1:** Age distribution for the Queensland Social Survey 2014 compared with census data [31,32].

Age	QSS 2014	Census QLD	Difference
18–24	3.2%	12.5%	-9.3%
25–34	4.6%	17.9%	-13.3%
35–44	12.6%	18.9%	-6.3%
45–54	20.7%	18.0%	2.7%
55–64	24.9%	15.3%	9.6%
65+	33.6%	17.4%	16.2%

In response to “willingness to consult a veterinarian at their own expense” 79.8% (95% CI 81.96%-77.46%) (976/1223) were willing, while 10.2% (125/1223) were not willing and 7.9% (97/1223) would consider it, but would not be willing to do it; 21 respondents were unsure. Of those who were unwilling to consult a veterinarian at their own expense, 59.9% (95% CI 66.41%-53.14%) (133/222) said they would be willing if it was funded by Medicare while 36.5% (81/222) said they would not be willing even if funded by Medicare; eight respondents were unsure or did not respond. Overall 90.7% (95% CIs 92.18%-88.92%) (1109/1223) respondents were willing to consult with a veterinarian on the advice of their doctor if the consultation was funded by Medicare.

The percentages of respondents aged between 25–54 years willing to be referred to a veterinarian were lower than for other years, but this was not significant. There was also no significant difference by gender, the two broad geographic regions, Australian citizenship status, level of education, work pattern in week prior, work status, industry of work, employment status, gross income per week, combined household gross income per week, and type of area lived in. Not all participants answered all questions (to work status and industry of work only 675 answered and to employment status only 548 answered).

Of those who were “willing if the veterinary consultation fee was funded under Medicare”, younger age categories (<45 years) were more willing to consult a veterinarian (Table 2). However, this was not significant. None of the variables listed above showed any significant difference for this question.

**Table 2:** Willingness to consult a veterinarian if funded by Medicare for those respondents who were not willing to do so at their own expense; Queensland Social Survey, 2014.

Age category	Yes	No	DK/NA	Total	Percent
18–24 yrs (1)	4	0	0	4	100.0%
25–34 yrs (2)	9	2	0	11	81.8%
35–44 yrs (3)	22	9	0	31	71.0%
45–54 yrs (4)	34	22	3	59	57.6%
55–64 yrs (5)	23	21	2	46	50.0%
>65 yrs (6)	41	26	3	70	58.6%
Overall	133	81	8	222	59.9%

## Discussion

The survey found that a large majority of Queensland residents were willing to consult a veterinarian for a zoonotic disease if their physician recommended it. Surprisingly, the majority reported that they would consult a veterinarian even if they had to bear the cost themselves. Having the consultation funded by the government health insurance scheme increased the proportion willing to consult a veterinarian to 90.7%. Price sensitivity was minimal. It seems that the Australian public may be strongly in favor of this novel referral pathway.

General practitioner (GP) referrals to allied health professionals with the purpose of improving treatment outcomes are a well-established practice and are formally recognized by health departments and insurance bodies. For example, Australia’s public health insurance scheme, Medicare, currently lists 13 allied health professions as eligible providers for specified patients with chronic or terminal conditions [34]. These disciplines fall into two categories: standard health disciplines (Aboriginal Health Workers or Aboriginal and Torres Strait Islander Health Practitioners, audiologists, diabetes educators, dieticians, exercise physiologists, mental health workers, occupational therapists, physiotherapists, podiatrists, psychologists and speech pathologists) and complementary medicine practitioners (chiropractors, osteopaths). Recognition of the eligibility to act as providers depends largely on credentialing by the respective professional body [34]. Integrating GP and allied health care improved patient outcomes and patient satisfaction and increased communication between GPs and allied health professionals [35,36].

Formalizing the professional relationship between physicians and veterinarians may already have some level of support from both professions. Using the case of people with AIDS, a group at higher risk of zoonoses, veterinarians provided advice to HIV/AIDS patients on zoonotic diseases more frequently than physicians did [12,13]. The majority of physicians and veterinarians thought both professions should be involved in discussing zoonoses with patients [12,13]. Physicians also thought that veterinarians were more knowledgeable about zoonoses than their own profession [11,13]. Veterinarians have multi-species training and experience and are more familiar than physicians in considering management strategies involving more than one species [37]. Support for a formal role of veterinarians in the management of individual cases of zoonotic disease should be specifically explored.

The limitations of this survey are the usual limitations of a CATI, such as the under-representation of younger age groups. Response rates to general household surveys conducted by CATI have fallen over the years, younger people are less likely to take part, but the response rate obtained with the QSS 2014 is typical of current rates [38,39]. Unfortunately, we are unable to make any comment about the differences between those who answered the CATI and those who refused. However, it would be surprising if the refusals were so markedly different as to reduce the rate of support for question 1 and 2 combined below 50% (<27% in refusals versus 91% in participants). The QSS is conducted via land-line and does not call mobile phones. This may explain the bias towards older age groups.

This study is only one of several that need to be conducted to gauge the possibilities for physicians and veterinarians to formally collaborate in managing zoonoses in the clinical situation. The expectations and attitudes of both physicians and veterinarians to this proposal must be explored. Options for implementation of this style of collaborative care in various settings need to be examined and discussed. The logistical, administrative and policy challenges to implementing this strategy should be investigated with professional bodies, health departments and health insurers.

In addition we anticipate that bringing physicians and veterinarians together in managing patients would improve communication between the professions and catalyze further practical applications under Clinical One Health, improving clinical practice in both disciplines [10].
